# Supplementary material for: A density-based enrichment measure for assessing colocalization in single-molecule localization microscopy data
Source: Nat Commun. 2022 Jul 28;13:4388. doi: 10.1038/s41467-022-32064-y (PMC9334352; doi:10.1038/s41467-022-32064-y)
Supplement: Supplementary file 1 — Supplementary Information [file 41467_2022_32064_MOESM1_ESM.pdf]

A density-based enrichment measure for assessing colocalization in single-molecule localization microscopy data

Ejdrup et al.

Supplementary information

Supplementary Figures 1-5

Supplementary Figure 1

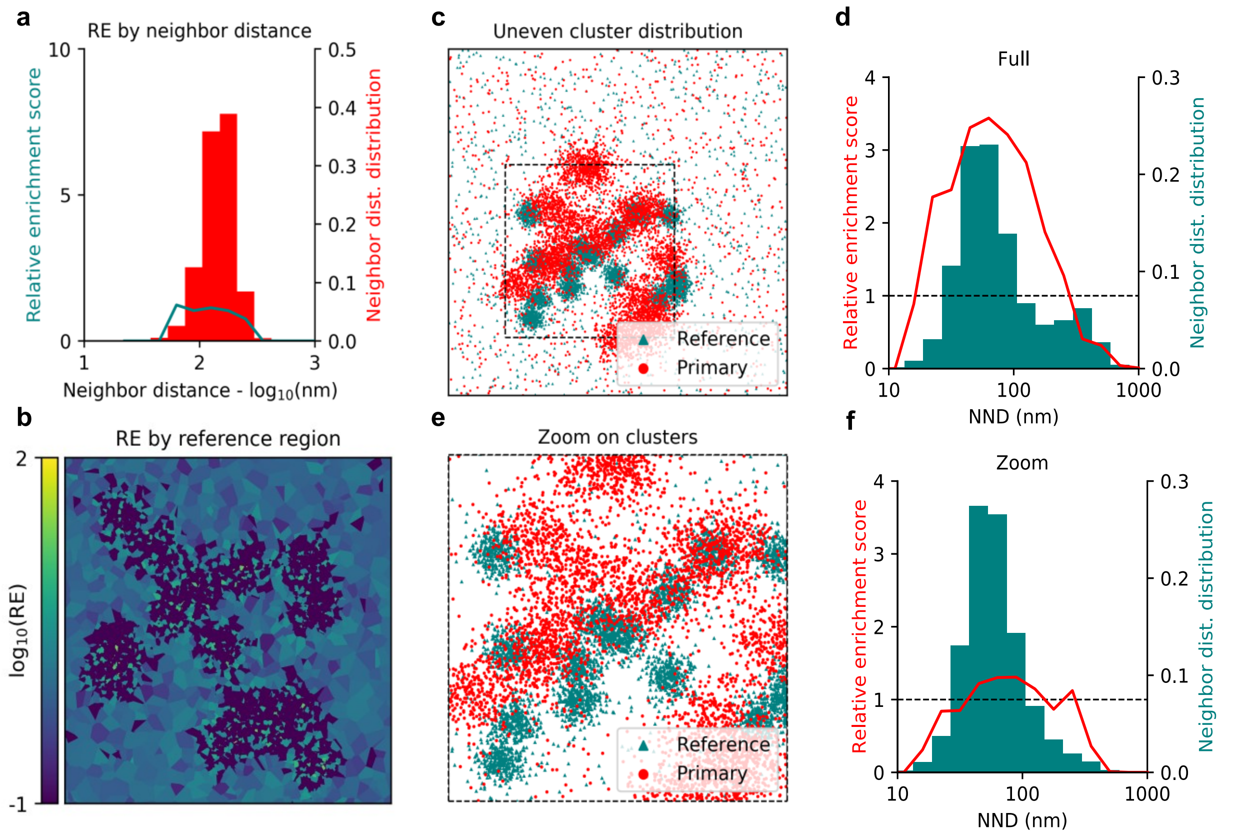

Supplementary Figure 1. **Uneven cluster distribution. a** Mean RE score across reference densities, binned by nearest neighbour distance (NND), for data on Fig. 2g, but with the species reversed. The narrow reference histogram reflects the uniform distribution. **b** Voronoï regions for the reference species, color-coded by their individual RE scores, calculated based on the randomly distributed primary species. **c** Simulation of two species, with a clustered fraction in both species confined to the same area. **d** Mean RE score across reference densities of the entire area in (**c**). **e** Zoom on the dashed area in (**c**). **f** Mean RE score across reference densities of the zoom on (**e**).

Supplementary Figure 2

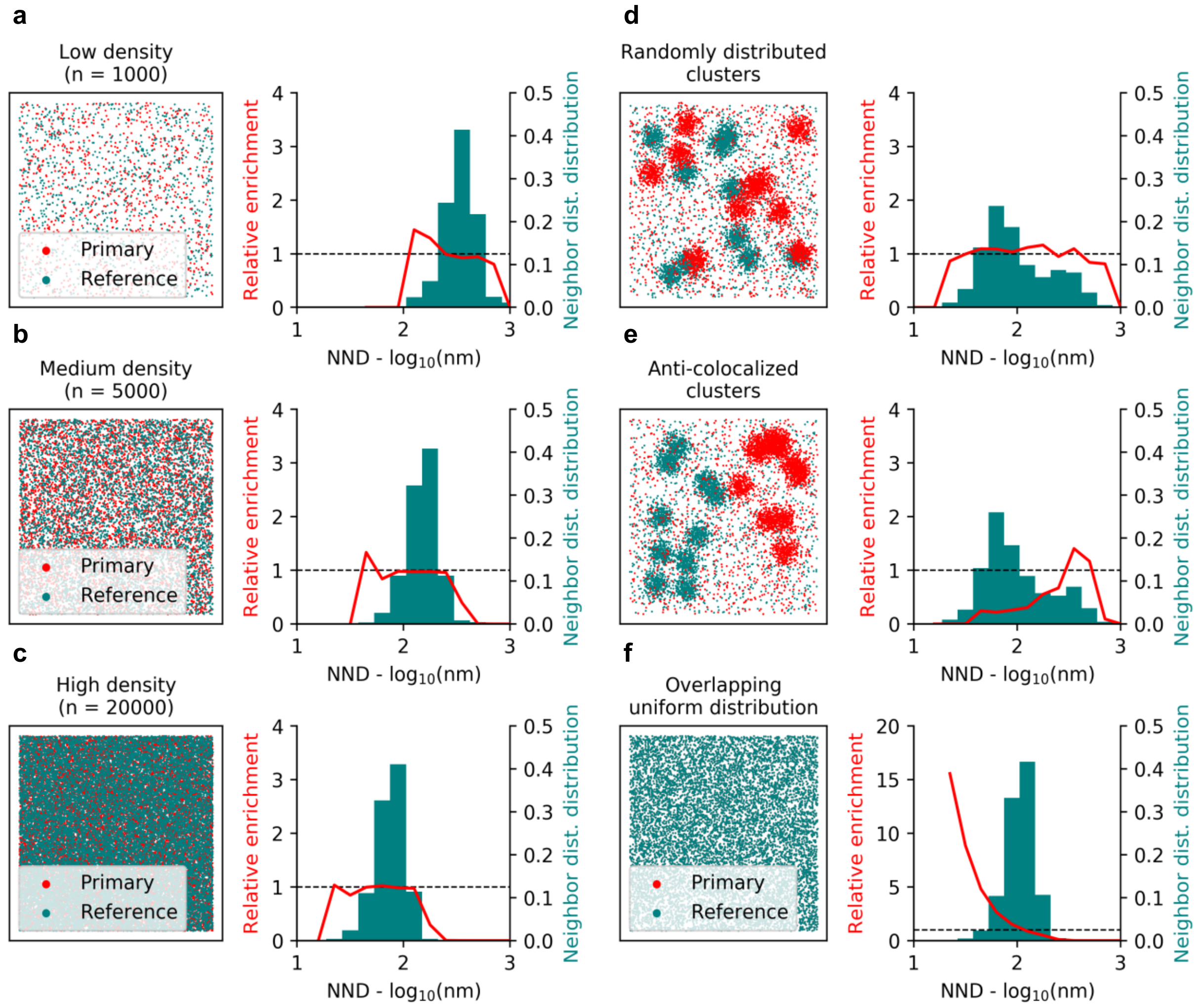

Supplementary Figure 2. **Various simulated distributions. a** Two random uniform distributions with n = 1,000 and their resulting relative enrichment score. **b** Same as (**a**) but with n = 5,000. **c** Same as (**a**) but with n = 20,000. **d** Two populations with randomly distributed clusters and background noise and the resulting relative enrichment score. **e** Same as (**d**), but with anti-colocalizing clusters. **f** Two perfectly colocalizing species from the same random uniform distribution. The resulting relative enrichment score is the inverse of the mean area of the density bin. Dashed line indicates RE of 1 in (**a**-**f**).

Supplementary Figure 3

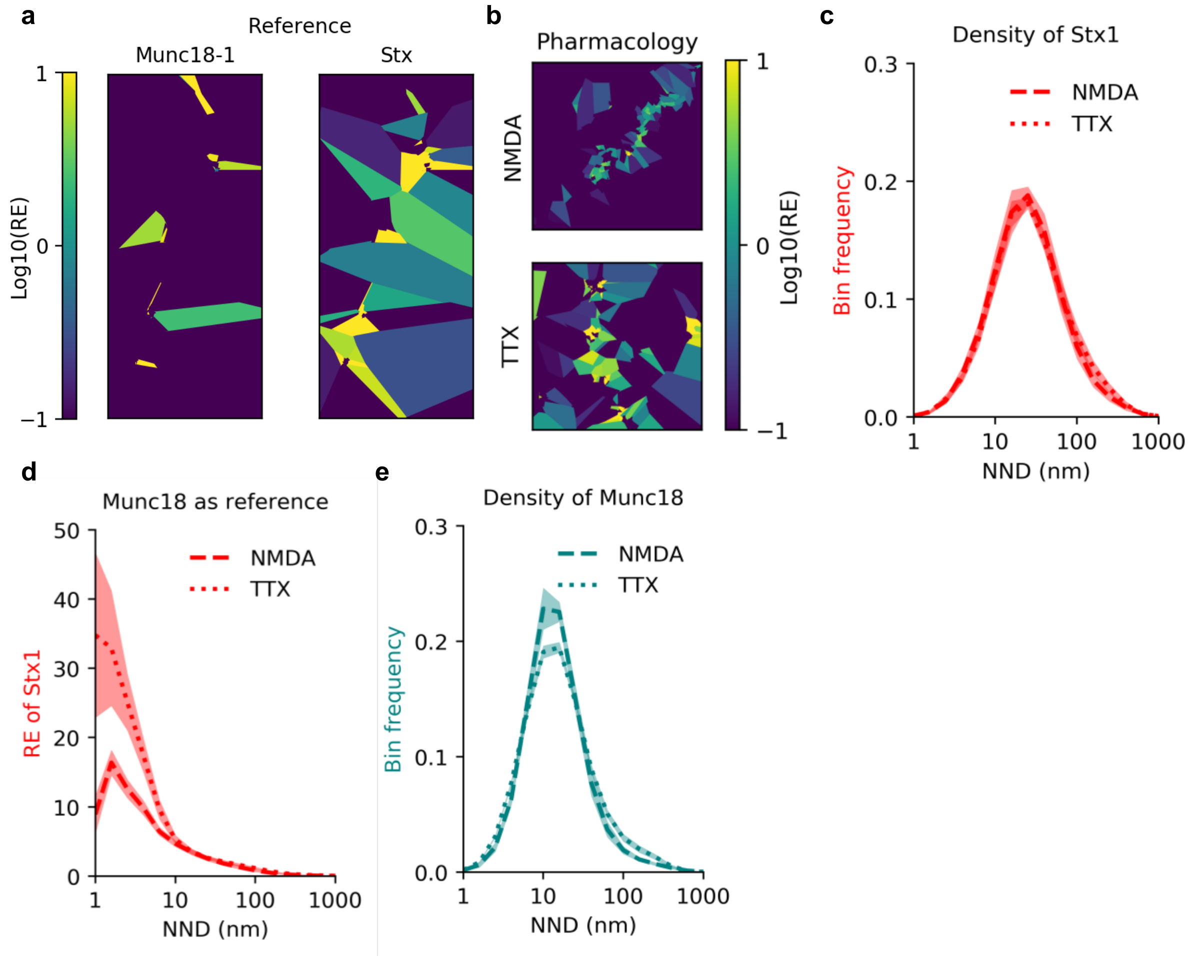

Supplementary Figure 3**. Munc18-1 and Stx1 supplemental data. a** Voronoï regions of Fig. 3C color-coded for RE values. **b** Voronoï regions of Fig. 3G color-coded for RE values. **c** Density of Stx1 after NMDA (dashed, n = 7 images) or TTX (dotted, n = 8 images) treatment. Shaded area indicates S.E.M.. **d** Relative enrichment of Stx1 across Munc18-1 densities after NMDA (dashed, n = 7 images) or TTX (dotted, n = 8 images) treatment. Shaded area indicates S.E.M.. **e** Density of Munc18-1 after NMDA (dashed, n = 7 images) or TTX (dotted, n = 8 images) treatment. Shaded area indicates S.E.M..

Supplementary Figure 4

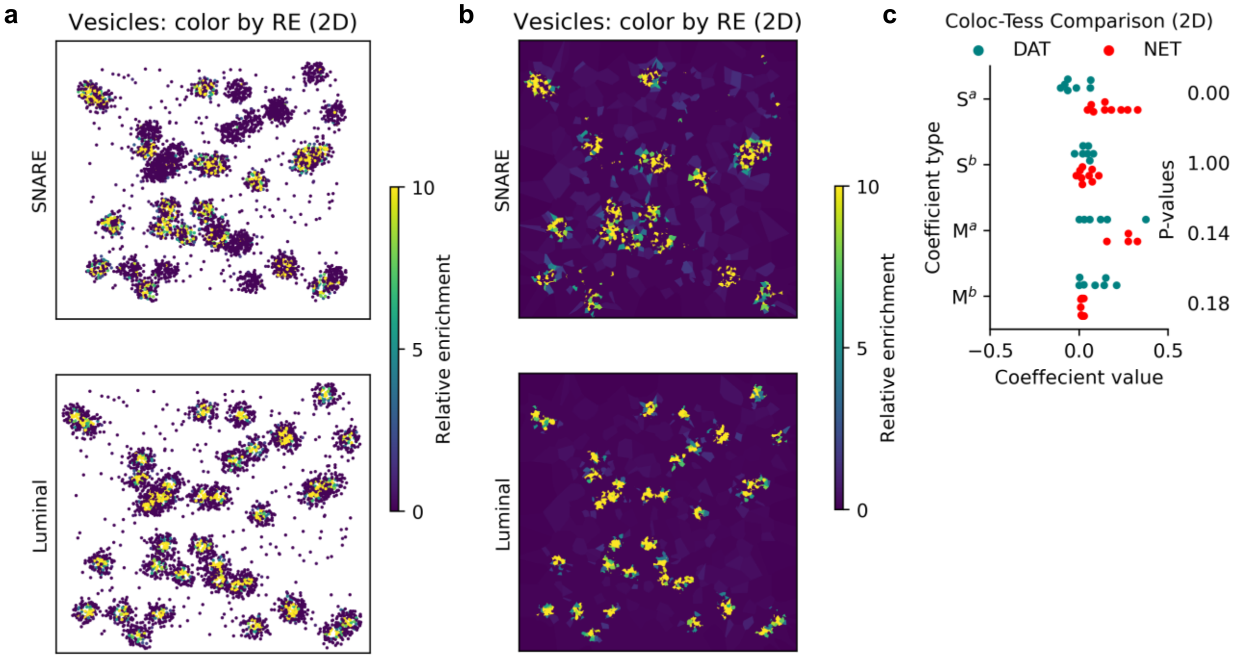

Supplementary Figure. 4**. Color-coding of simulated vesicular species. a** Individual localization of the vesicle species color-coded for RE score of either the AZ (top) or cargo (bottom) species. **b** Same as (**a**), but with color-coded Voronoï regions. **c** Spearman (S) and Mander's (M) coefficient computed in 2D as per [12] (H_0: DAT = NET. S^{EEA1}, p = 0.00; $S^{D/N}$, p = 1.00; M^{EEA1}, p = 0.15; $M^{D/N}$, p = 0.18. Unpaired, one-sided student's t-test by image, FWER correction with Bonferroni-Holm).

Supplementary Figure 5

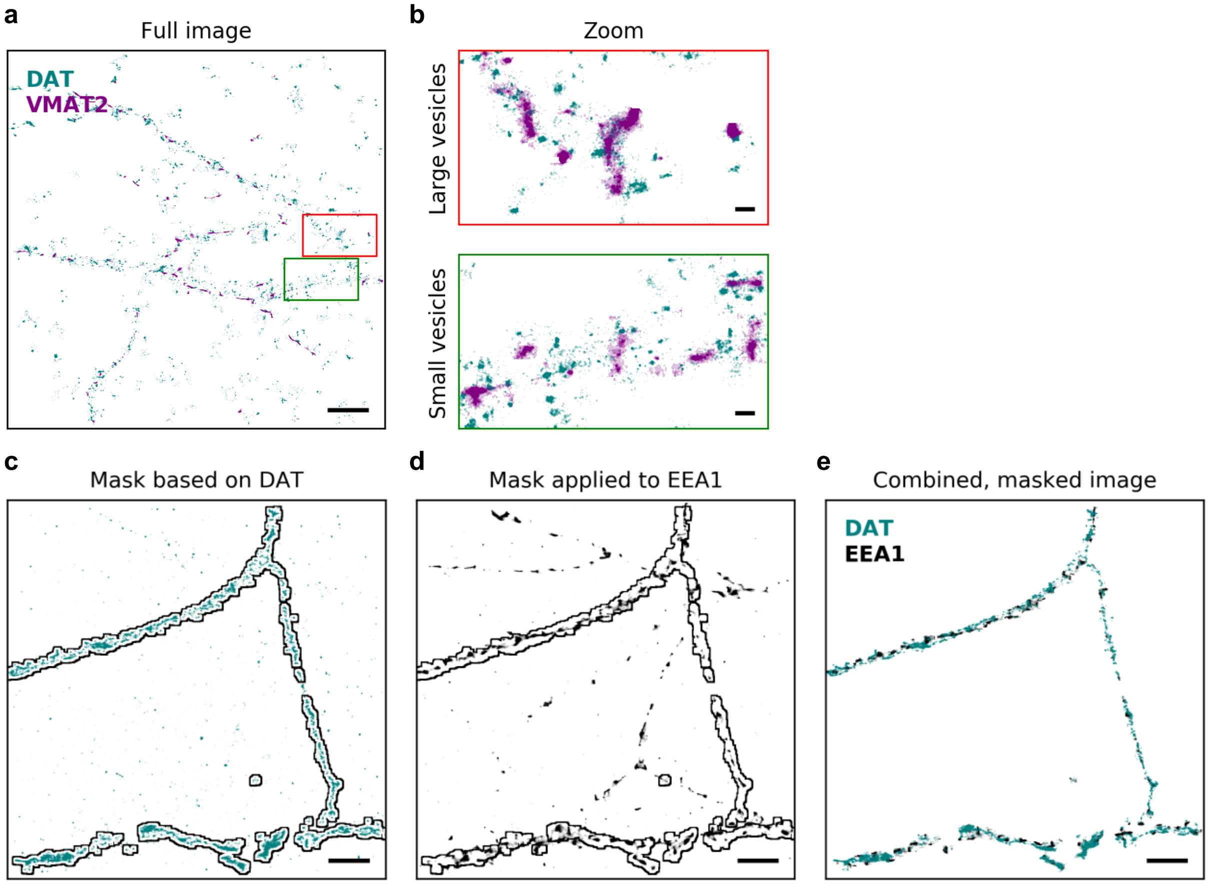

Supplementary Figure 5. **VMAT2-positive vesicle sizes and masking of dopaminergic neurons.** **a** Representative top view of image with VMAT2 and DAT from primary midbrain cultures acquired with astigmatic dSTORM. Molecular localizations visualized by gaussian representation. Scale bar is 4 µm. **b** Zoom on red and green inset in (**a**) showing varicosities with putative large dense-core vesicles (top) and synaptic vesicles (bottom). Scalebars are 200 nm. **c** Representative top view of DAT-expression in primary midbrain cultures automatically masked to identify extensions. Scale bar is 4 µm. **d** Mask from (**c**) applied to EEA1-staining of

the same culture to extraction DAergic-specific expression. Scale bar is 4 µm. **e** Combination of DAT and EEA1 stain masked based on (**c**). Scale bar is 4 µm.
