# Supplementary material for: A density-based enrichment measure for assessing colocalization in single-molecule localization microscopy data
Source: Nat Commun. 2022 Jul 28;13:4388. doi: 10.1038/s41467-022-32064-y (PMC9334352; doi:10.1038/s41467-022-32064-y)
Supplement: Supplementary file 3 — Reporting Summary [file 41467_2022_32064_MOESM3_ESM.pdf]

nature portfolio

Corresponding author(s): Aske L. Ejdrup, Ulrik Gether.

Last updated by author(s): Jul 14, 2022

Reporting Summary

Nature Portfolio wishes to improve the reproducibility of the work that we publish. This form provides structure for consistency and transparency in reporting. For further information on Nature Portfolio policies, see our Editorial Policies and the Editorial Policy Checklist.

Statistics

For all statistical analyses, confirm that the following items are present in the figure legend, table legend, main text, or Methods section.

n/a	Confirmed	
☐	☒	The exact sample size (n) for each experimental group/condition, given as a discrete number and unit of measurement
☐	☒	A statement on whether measurements were taken from distinct samples or whether the same sample was measured repeatedly
☐	☒	The statistical test(s) used AND whether they are one- or two-sided<br>*Only common tests should be described solely by name; describe more complex techniques in the Methods section.*
☐	☒	A description of all covariates tested
☐	☒	A description of any assumptions or corrections, such as tests of normality and adjustment for multiple comparisons
☐	☒	A full description of the statistical parameters including central tendency (e.g. means) or other basic estimates (e.g. regression coefficient) AND variation (e.g. standard deviation) or associated estimates of uncertainty (e.g. confidence intervals)
☐	☒	For null hypothesis testing, the test statistic (e.g. F, t, r) with confidence intervals, effect sizes, degrees of freedom and P value noted<br>*Give P values as exact values whenever suitable.*
☒	☐	For Bayesian analysis, information on the choice of priors and Markov chain Monte Carlo settings
☒	☐	For hierarchical and complex designs, identification of the appropriate level for tests and full reporting of outcomes
☒	☐	Estimates of effect sizes (e.g. Cohen's d, Pearson's r), indicating how they were calculated

Our web collection on statistics for biologists contains articles on many of the points above.

Software and code

Policy information about availability of computer code

Data collection	Detailed descriptions of image acquisition are written in the method section of the paper.
Data analysis	All custom python functions and working examples with data from the simulated 2D and 3D vesicle example are available online at: https://github.com/Ejdrup/relative-enrichment/releases/tag/v0.1.0 or at DOI: 10.5281/zenodo.6627703. The figure-specific analyses are available from the corresponding author within two weeks upon reasonable request.<br><br>Statistical analyses were carried out in Python 3.6.10 with the open-source python packages SciPy v1.5.2, Numpy v1.18.1, and Seaborn v0.11.0, and linear models in Statsmodels v0.12.2.

For manuscripts utilizing custom algorithms or software that are central to the research but not yet described in published literature, software must be made available to editors and reviewers. We strongly encourage code deposition in a community repository (e.g. GitHub). See the Nature Portfolio guidelines for submitting code & software for further information.

Data

Policy information about availability of data

All manuscripts must include a data availability statement. This statement should provide the following information, where applicable:

- Accession codes, unique identifiers, or web links for publicly available datasets
- A description of any restrictions on data availability
- For clinical datasets or third party data, please ensure that the statement adheres to our policy

The processed data generated in this study are provided in the Source Data file. Due to size the raw dSTORM images are not included, but available from the corresponding author within two weeks upon reasonable request.

Field-specific reporting

Please select the one below that is the best fit for your research. If you are not sure, read the appropriate sections before making your selection.

☒ Life sciences ☐ Behavioural & social sciences ☐ Ecological, evolutionary & environmental sciences

For a reference copy of the document with all sections, see nature.com/documents/nr-reporting-summary-flat.pdf

Life sciences study design

All studies must disclose on these points even when the disclosure is negative.

Sample size	We performed no sample size calculations prior to the experiments due to unknown effect sizes. We aimed for a minimum of five samples based on a priori experience with this type of experiments and to minimize the number of sacrificed animals.
Data exclusions	We excluded captured dSTORM videos where the focus was too poor to perform efficient fits with 3D-DAOSTORM.
Replication	Transporter internalizations experiments were repeated three times with the same outcome. Reported statistics are for pooled data. Hippocampal synapse organization experiments were performed with a pool of dissected neurons from one set of animals, but on three separete sets of prepared cultures that all showed the same result. Further dissections were not performed to reduce animal use as sufficient statistical power was obtained. Reported statistics are from pooled data.
Randomization	All experiments contained multiple conditions. For all experiments the samples were imaged in alternating order to account for buffer and sample deterioration. Conditions were mixed across sample plates and randomly assigned.
Blinding	Experiments were not performed blinded, as the resolution necessary to resolve the nanoarchitecture is not obtained until after pre-processing. Therefore the experimenter is blinded to the result during imaging. All pre-processing was done with an automated pipeline with human input only for setting a standard deviation threshold for peak detection on the level of the entire experiment.

Reporting for specific materials, systems and methods

We require information from authors about some types of materials, experimental systems and methods used in many studies. Here, indicate whether each material, system or method listed is relevant to your study. If you are not sure if a list item applies to your research, read the appropriate section before selecting a response.

Materials & experimental systems

n/a	Involved in the study	
☐	☒	Antibodies
☐	☒	Eukaryotic cell lines
☒	☐	Palaeontology and archaeology
☐	☒	Animals and other organisms
☒	☐	Human research participants
☒	☐	Clinical data
☒	☐	Dual use research of concern

Methods

n/a	Involved in the study	
☒	☐	ChIP-seq
☒	☐	Flow cytometry
☒	☐	MRI-based neuroimaging

Antibodies

Antibodies used

Anti-Stx1, Synaptic Systems, 110 011; Anti-Munc18-1, Synaptic Systems, 116 002; Anti-DAT, Sigma-Aldrich, MAB369; Anti-hNET, MAb Technologies, NET17-1; Anti-VMAT2, from Professor Garry Miller; Anti-EEA1, Abcam, Ab2900; Anti-mouse AF568, ThermoFisher, A-11004, Anti-mouse AF568, ThermoFisher, A-11004; Anti-rabbit, AF568, ThermoFisher, A-11011; Anti-rabbit AF647, ThermoFisher, A-21245; Anti-rat AF647, ThermoFisher, A-21247; Anti-mouse AF647, ThermoFisher, A-21235.

Validation

Validation of the used antibodies are specified either on the suppliers website or in the papers explicitly stated:

Primary antibodies:
Anti-Stx1, Synaptic Systems, 110 011: https://sysy.com/product/110011
Anti-Munc18-1Synaptic Systems, 116 002: https://sysy.com/product/116002
Anti-DAT, Sigma-Aldrich, MAB369: https://www.sigmaaldrich.com/product/mm/mab369, and in-house validation (DOI: 10.1016/j.jbc.2021.101361)
Anti-hNET, MAb Technologies, NET17-1: https://mabtechnologies.com/categories/product/5-norepinephrine-transporter-human-net17-1, and in-house validation (DOI: 10.1074/jbc.M115.702050)
Anti-VMAT2, developed and gifted by Professor Garry Miller. See original publication, Cliburn et al., J Chem Neuroanat. (2017), and further validation Rahbek-Clemmensen et al., Nat Comms (2017).
Anti-EEA1, Abcam, Ab2900: https://www.abcam.com/eea1-antibody-early-endosome-marker-ab2900.html.

Secondary antibodies:
Anti-mouse AF568, ThermoFisher, A-11004: https://www.thermofisher.com/antibody/product/Goat-anti-Mouse-IgG-H-L-Cross-Adsorbed-Secondary-Antibody-Polyclonal/A-11004.
Anti-rabbit, AF568, ThermoFisher, A-11011: https://www.thermofisher.com/antibody/product/Goat-anti-Rabbit-IgG-H-L-Cross-Adsorbed-Secondary-Antibody-Polyclonal/A-11011.
Anti-rabbit AF647, ThermoFisher, A-21245: https://www.thermofisher.com/antibody/product/Goat-anti-Rabbit-IgG-H-L-Highly-Cross-Adsorbed-Secondary-Antibody-Polyclonal/A-21245.
Anti-rat AF647, ThermoFisher, A-21247: https://www.thermofisher.com/antibody/product/Goat-anti-Rat-IgG-H-L-Cross-Adsorbed-Secondary-Antibody-Polyclonal/A-21247.
Anti-mouse AF647, ThermoFisher, A-21235: https://www.thermofisher.com/antibody/product/Goat-anti-Mouse-IgG-H-L-Cross-Adsorbed-Secondary-Antibody-Polyclonal/A-21235.

Eukaryotic cell lines

Policy information about cell lines

Cell line source(s)	PC-12, ATCC: CRL-1721
Authentication	The cell line was not authenticated.
Mycoplasma contamination	All the cells were tested negative for mycoplasma infection.
Commonly misidentified lines (See ICLAC register)	None

Animals and other organisms

Policy information about studies involving animals; ARRIVE guidelines recommended for reporting animal research

Laboratory animals	Wistar rat, E19 for hippocampal cultures and P1-P3 for dopaminergic cultures, all of mixed gender (Charles River, Wilmington, MA).
Wild animals	No wild animals were used.
Field-collected samples	No field-collected samples were used in this study.
Ethics oversight	All animal experiments were approved by the Danish Animal Experiments Inspectorate. All efforts were made to minimize animal suffering and to reduce the number of animals used.

Note that full information on the approval of the study protocol must also be provided in the manuscript.
